# Handle Region Peptide Ameliorating Insulin Resistance but Not ****β**** Cell Functions in Male Rats Neonatally Treated with Sodium L-Glutamate

**DOI:** 10.1155/2013/493828

**Published:** 2013-12-10

**Authors:** Guo-shu Yin, Shao-da Lin, Dong-chuan Xu, Ru-qiong Sun, Kun Lin, Chu-jia Lin

**Affiliations:** Department of Endocrinology and Metabolism, The First Affiliated Hospital of Shantou University Medical College, 57 Changping Road, Shantou, Guangdong 515041, China

## Abstract

Handle region peptide (HRP), which was recognized as a blocker of (pro)renin receptor ((P)RR), may block the function of (P)RR. The aim of this study was to investigate the effect of HRP with a large dose of 1 mg/kg/d on glucose status in the rats treated neonatally with monosodium L-glutamate (MSG). At the age of 8 weeks, the MSG rats were randomly divided into MSG control group, HRP treated group with minipump (MSG-HRP group), losartan treated group (MSG-L group), and HRP and losartan cotreated group (MSG-HRP-L group) and fed with high-fat diet for 4 weeks. Losartan but not HRP increased the levels of insulin releasing and ameliorate glucose status although both losartan and HRP improved insulin sensitivity. On the one hand, both losartan and HRP decreased levels of pancreatic local Ang-II and NADPH oxidase activity as well as its subunits P^22phox^. On the other hand, losartan but not HRP decreased **α**-cell mass and number of PCNA-positive cells located periphery of the islets and decreased picrosirius red stained area in islets. HRP ameliorating insulin resistance but not **β**-cell functions leads to hyperglycemia in the end in male MSG rats, and the dual characters of HRP may partly account for the phenomenon.

## 1. Introduction

Recent clinical studies have shown that treatment with angiotensin II type 1 (AT1) receptor blockers or angiotensin-converting enzyme (ACE) inhibitors protects against the development of insulin resistance in hypertensive patients and new onset of diabetes in “at-risk” patients, indicating that the renin-angiotensin system (RAS) especially tissue RAS may contribute to the regulation of glucose metabolism [1–4]. These functional local RASs have been found in diverse organ systems [5] such as the pancreas [6–8], heart [9, 10], kidney [11], vasculature [12], adipose tissue [13, 14], and skeletal muscle [15].

The (pro)renin receptor ((P)RR) cloned in 2002 was reported to be expressed in various tissues [6]. Its ligand, prorenin, is known to be activated without catalytic conversion into mature renin when combining with the (P)RR and alters the activation of extracellular signal-regulated kinase 1/2 (ERK1/2) [6, 7, 16, 17]. Although aliskiren theoretically will block such prorenin-dependent Ang generation, an alternative way to suppress this Ang source is the infusion of the (P)RR blocker called the handle region peptide (HRP). Its effectiveness in vivo is controversial, in part because of a wide variety of doses that has been applied, ranging from 0.1 mg/kg per 28 days to 1.0 mg/kg per day [18, 19].

Moreover, the monosodium glutamate- (MSG-) induced obese rat is a model associated with insulin resistance that may occur without the presence of type 2 diabetes, depending on the age at which the animals are studied. The administration of MSG to newborn rats results in distinctive lesions in hypothalamic arcuate nucleus (ARC) neurons. The neuronal loss impairs insulin and leptin signaling and impacts energy balance as well as pituitary and adrenal activity [20].

This study aimed to determine the effect of HRP with a large dose of 1 mg/kg/d on glucose status in the MSG rats.

## 2. Methods

### 2.1. Animals

All animal protocols were approved by the Ethics Committee of Shantou University Medical College. Timed pregnant Sprague-Dawley rats were obtained from Laboratory Animal Center of Shantou University Medical College. Neonatal male rats were injected subcutaneously with either 4 mg/g of MSG (Sigma Aldrich, Mo, USA) or NaCl (1.87% solutions) as control. All of the animals were ablactated at 3 weeks age. The control group of rats was given normal diet, whereas all of the MSG rats were given high-energy diet (445.5 Kcal/100 g, Slaccas, Shanghai, China). At age of 8 weeks, the MSG-treated rats were divided into 4 groups including the MSG-control group (MSG group, *n* = 6), HRP treated group (MSG-HRP group, *n* = 6), losartan treated group (MSG-L group, *n* = 6), and losartan and HRP cotreated group (MSG-HRP-L group, *n* = 6). Then (day 0 and day 15) osmotic minipumps (2ML4 ALZET, CA, USA) were implanted subcutaneously under isoflurane anesthesia to infuse vehicle (saline) or HRP (NH2-RILLKKMPSV-COOH, Chinapeptides, Shanghai, China, 1 mg/kg per day, for the MSG-HRP and MSG-HRP-L group). The rats of MSG-L group and MSG-HRP-L group were given drinking water with 0.45 g/L losartan (Merck, Hangzhou, China) [21].

### 2.2. Oral Glucose Tolerance Test (OGTT) and Insulin Tolerance Test (ITT)

Oral glucose tolerance test (OGTT) was performed after 16 h overnight fasting. Glucose (2 g/kg) was administered orally, and a small amount of blood (about 100 mL) was collected from the tail vein at 0, 30, 60, and 120 min for insulin (ELISA, Cusabio, Wuhan, China) and glucose measurement immediately with a glucometer (Johnson & Johnson, New Brunswick, USA). For insulin tolerance test (ITT), after 4 h of fasting, rats were given an intraperitoneal injection of 0.5 U/kg human insulin (Novo Nordisk, Tianjin, China), and glucose was measured immediately with a glucometer at 0, 15, 30, and 90 min.

### 2.3. Systolic Blood Pressure (SBP)

Restraint conditioning was initiated before blood pressure measurements. SBP was measured in triplicate on separate occasions throughout the day, using the tail-cuff method (Kent Scientific Copporartion, Connecticut, USA) before the animals were sacrificed.

### 2.4. Measurement of Physiological and Biochemical Parameters

At 12 weeks of age, body weight and length were measured, and Lee's index was calculated according to the formula: Lee's index = $\sqrt[{3}]{\text{Weight}\;(\text{g})}\times 1000/\text{Length}\;(\text{cm})$. After the performance of OGTT and ITT, rats were sacrificed using pentobarbital sodium with the dose of 50 mg/kg weight. Blood samples from puncturing heart were collected into EDTA tubes for the measurement of plasma Ang-II concentration. The pancreas was rapidly dissected out and bisected longitudinally, with one half snap frozen in liquid nitrogen and stored at −80°C before use, and the other half fixed in 4% paraformaldehyde and embedded in paraffin.

### 2.5. Picrosirius Red Staining

Four-micron paraffin sections were prepared from 4% paraformaldehyde-fixed, paraffin-embedded rat pancreas. Sections were stained with 0.1% sirius red (Sigma Aldrich, Mo, USA) in saturated picric acid (picrosirius red) for 1 h and mounted. The ratio of stained area to the area of whole islet was calculated using the computer-imaging software IPP6.0.

### 2.6. *β*-Cell Mass and *α*-Cell Mass

The expressions of the *β*-cell marker insulin and *α*-cell marker glucagon were examined by immunohistochemistry using insulin antibody (1 : 1,000; Santa Cruz, TX, USA) and glucagon antibody (1 : 100; Santa Cruz, TX, USA), respectively. Slides were incubated with the primary antibody for 1 h at room temperature. After washing, a secondary antibody (1 : 500, biotin-conjugated goat anti-rabbit IgG; Boster, Wuhan, China) was applied for 30 min at room temperature. The ratio of stained area (*β*-cell mass and *α*-cell mass) to the area of whole islet was calculated using IPP6.0. The values were obtained from three islets in each section obtained from six rats in each group.

### 2.7. Islet Cell Proliferation

Proliferation of intraislet was assessed by immunohistochemical staining for proliferating cell nuclear antigen (PCNA) using PCNA antibody (1 : 100; Santa Cruz, TX, USA). Specific immunohistochemical staining was detected using the streptavidin horseradish peroxidase and DAB as the chromogen. Semiquantitative assessment of intraislet proliferation was performed by determining the number of PCNA-positive cells per islet section.

### 2.8. Assay of NADPH Oxidase Activity and Subunit of NADPH Oxidase

Pancreatic tissue was obtained and homogenized with Tris-HCl buffer (pH 7.0). NADPH oxidase activity was assayed using cytochrome C (GENMED, Shanghai, China). For assessing subunit of NADPH oxidase P^22phox^ in islet sections, staining was performed for P^22phox^ (1 : 100; Santa Cruz, TX, USA). Slides were incubated with the primary antibody for 1 h at room temperature. After washing, a secondary antibody (1 : 500, biotin-conjugated goat anti-rabbit IgG; Boster, Wuhan, China) was applied for 30 min at room temperature. The average gray-scale intensities of cells staining positively were measured by IPP 6.0.

### 2.9. Local Pancreatic Ang-II Measurement

About 100 mg pancreas tissue was homogenized in 50 mmoL/L Tris buffer (pH 7.4), 150 moL/L NaCl, 1% Triton X-100, 1% sodium deoxycholate, 0.1% SDS, and some inhibitors with a homogenizer on the ice and then centrifuged at 12000 rpm for 15 min at 4°C. The resulting supernatants were collected. Protein concentrations were determined using the BCA method. The concentration of Ang-II was measured by ELISA (Cusabio, Wuhan, China) and the results were corrected by the protein concentration.

### 2.10. Statistical Analysis

Data are shown as means ± SD. ANOVA and DSL-test were performed to estimate differences between groups. Pearson correlation analysis was used to determine the relationship between variables. A value of *P* < 0.05 was considered statistically significant.

## 3. Results

### 3.1. Description of the Sample

The basal characteristics of each group are shown in the Table 1. Body weight was no statistical difference between Con group and MSG group. The MSG-L group and the MSG-HRP-L group had lower body weight than the MSG group (*P* < 0.05). Body length was decreased in the MSG group compared with the Con group and had no significant difference among MSG rats received different treatment. Lee's index and celiac adipose tissue wet weight, reflecting the extent of obesity, were increased in the MSG group compared with the Con group. The systolic blood pressure tended to increase in the MSG group but had no statistical difference. The MSG-L group and MSG-HRP-L group had obviously lower systolic blood pressure than the MSG group (*P* values were both <0.01). Serum Ang-II concentration had no significant difference between the Con group and the MSG group and was increased obviously in the MSG-HRP-L compared with the MSG group and the Con group (*P* values were both <0.01).

### 3.2. Glucose Status Measurement

**Figure 1:**
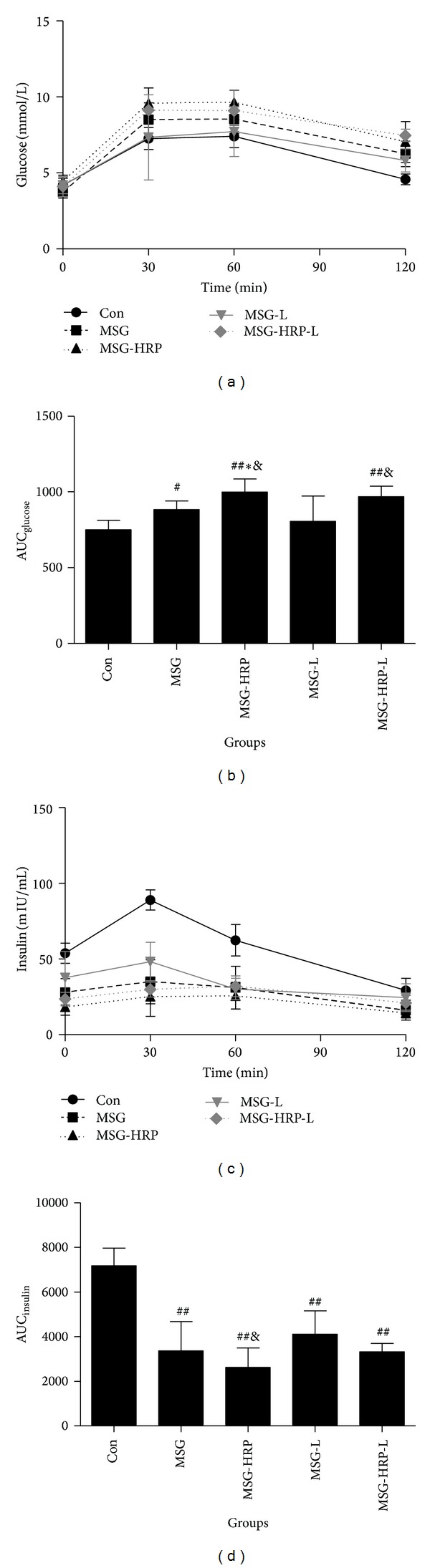
Samples for glucose (a) and serum insulin (c) before and 30 min, 60 min, and 120 min after glucose load were obtained and AUCs were calculated ((b) and (d), resp.). *Compared with MSG group, *P* < 0.05; **compared with MSG group, *P* < 0.01; ^#^compared with Con group, *P* < 0.05; ^##^compared with Con group, *P* < 0.01; ^&^compared with MSG-L group, *P* < 0.05.

The response of blood glucose to OGTT at week 12 of the experimental period was shown in Figures 1(a) and 1(b). Fasting blood glucose had no statistical difference among five groups of animals. The blood glucose was higher in the MSG-HRP group than the Con group at 30 min after glucose load. MSG group had no statistical difference with the MSG rats received any treatment. The blood glucose in the MSG-HRP group and the MSG-HRP-L group were both higher than the Con group at 60 min after glucose load (*P* values were <0.01 and 0.05, resp.) and MSG-HRP group was higher than the MSG-L group (*P* < 0.05). The blood glucose in the Con group was lower than the other four groups 2 h after glucose load and MSG-L group lower than the MSG-HRP group (*P* < 0.05). The area under the curve (AUC) of blood glucose in the Con group was lower than the MSG group, MSG-HRP group, and MSG-HRP-L group (*P* values were <0.05, <0.01, and <0.01, resp.) and had no statistical difference with the MSG-L group, whereas the MSG group was lower than the MSG-HRP group (*P* < 0.05) and the MSG-L group was lower than the MSG-HRP group and the MSG-HRP-L group (*P* values were both <0.05).

The response of serum insulin concentration to OGTT was shown in Figures 1(c) and 1(d). The serum insulin concentration of the Con group at fasting status, 30 minutes and 60 minutes after glucose load, and the AUC of insulin were higher than the other four groups. The AUC of insulin in the MSG-L group was higher than the MSG-HRP group (*P* < 0.05).

**Figure 2:**
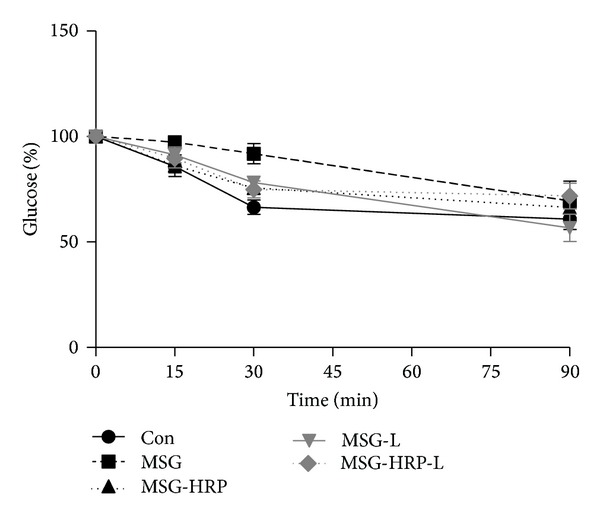
Insulin tolerance test was performed in all of the animals. The decreased rate of blood glucose was smaller in the MSG group compared with the Con group at 30 min after insulin injection. Treatment with HRP, losartan, and both HRP and losartan had higher decreased rate of blood glucose when compared with the MSG group.

Insulin sensitivity was evaluated according to ITT and the results were shown in Figure 2. The decreased rate of blood glucose was smaller in the MSG group compared with the Con group at 30 min after insulin injection (*P* < 0.01). Treatment with HRP, losartan, and both HRP and losartan had higher decreased rate of blood glucose when compared with the MSG group (*P* values were all <0.05).

### 3.3. *β*-Cell Mass and *α*-Cell Mass

**Figure 3:**
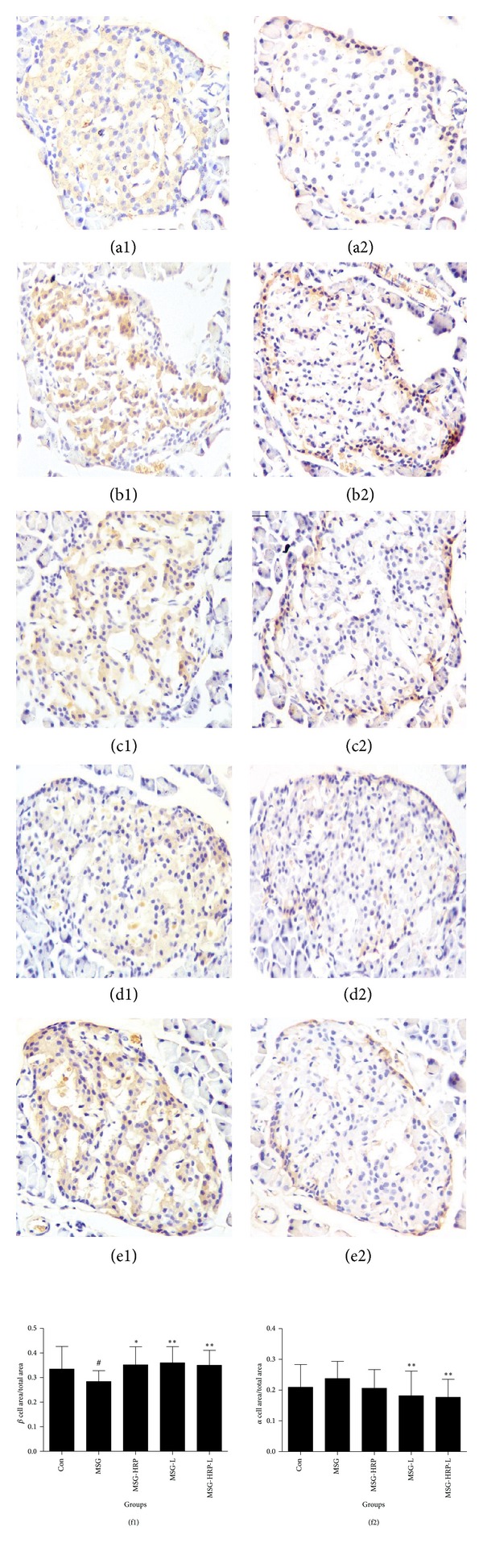
Insulin positive cells ((a1)–(e1)) and glucagon positive cells ((a2)–(e2)) in pancreatic islets (×400) and ratio of stained area (*β*-cell area and *α*-cell area) to the area of whole islet ((f1) and (f2)). (a) Con; (b) MSG; (c) MSG-HRP; (d) MSG-L; (e) MSG-HRP-L; *compared with MSG group, *P* < 0.05; **compared with MSG group, *P* < 0.01; ^#^compared with Con group, *P* < 0.05.

Islets *β* cell and *α* cell were marked by insulin antibody and glucagon antibody, respectively, according to immunohistochemistry. To quantify the change of *β*-cell mass and *α*-cell mass, the ratios of stained respective area of insulin and glucagon to the area of whole islet were calculated by IPP6.0 and the results were shown in Figure 3. The *β*-cell mass in pancreas islets was reduced in MSG rat when compared with Con group (*P* < 0.05). Treatment with HRP, losartan, and both increased *β*-cell mass when compared with the MSG group (*P* values were <0.05, <0.01, and <0.01, resp.). For the *α*-cell mass, MSG group and MSG-HRP group had no statistical difference. Treatment with losartan and both losartan and HRP reduced *α*-cell mass when compared with MSG group (*P* values were both <0.01).

### 3.4. Proliferation of Islets Cells

**Figure 4:**
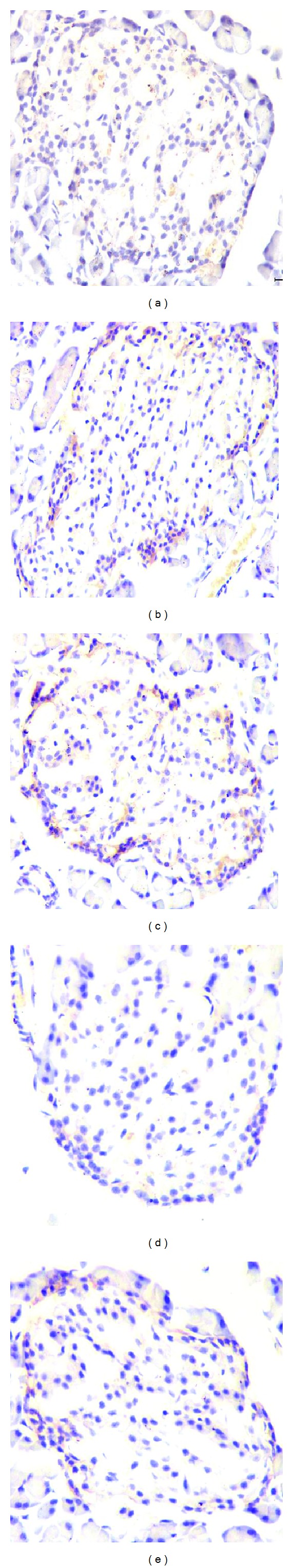
Cells staining positively for the PCNA marker in pancreatic islets (×400). Most PCNA-positive staining cells were distributed in the periphery of the islets and were in accordance with *α* cells. (a) Con; (b) MSG; (c) MSG-HRP; (d) MSG-L; (e) MSG-HRP-L.

Cells staining positively for the PCNA marker in pancreatic islets were shown in Figure 4. The number of the PCNA-positive staining cells was counted in the central area (70% of the central area of islet) and the peripheral area (30% area of the peripheral area of islets) of the islets according to IPP6.0. Most PCNA-positive staining cells were distributed in the periphery of the islets and were in accordance with the location of *α* cell. Compared with Con group, the number was increased in the MSG group (Con versus MSG: 12.15 ± 7.23 and 95.00 ± 9.04, *P* < 0.01). Treatment with HRP had not changed the number of PCNA-positive staining cells (90.25 ± 12.37), whereas treatment with losartan decreased the number obviously (22.92 ± 3.76, compared with MSG group, *P* < 0.01). Treatment with both HRP and losartan decreased the number obviously (43.35 ± 14.25, compared with MSG group, *P* < 0.01). There was no statistical difference for the PCNA-positive staining cells in the central of the islets.

### 3.5. Pancreatic Local Ang-II Levels

**Figure 5:**
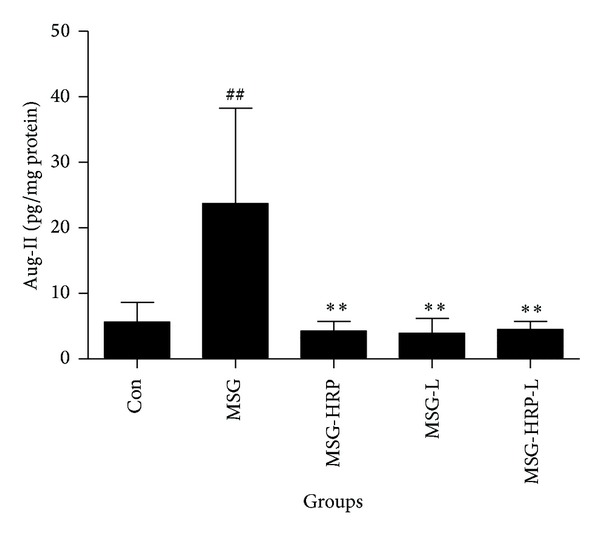
Levels of pancreas local Ang-II. Local Ang-II was obviously increased in the MSG group compared with the Con group. The MSG rats received treatment of HRP, losartan, and both HRP and losartan decreased the level of local Ang-II obviously compared with the MSG group (*P* values were all <0.01). **Compared with MSG group, *P* < 0.01; ^##^compared with Con group, *P* < 0.01.

The proteins of the pancreas tissue were extractted, and Ang-II was measured by ELISA (Figure 5). Pancreas local Ang-II was obviously increased in the MSG group compared with the Con group (*P* < 0.01). The MSG rats received treatment of HRP, losartan, and both HRP and losartan decreased the level of local Ang-II obviously compared with the MSG group (*P* values were all <0.01).

### 3.6. Fibrosis of the Pancreatic Islets

**Figure 6:**
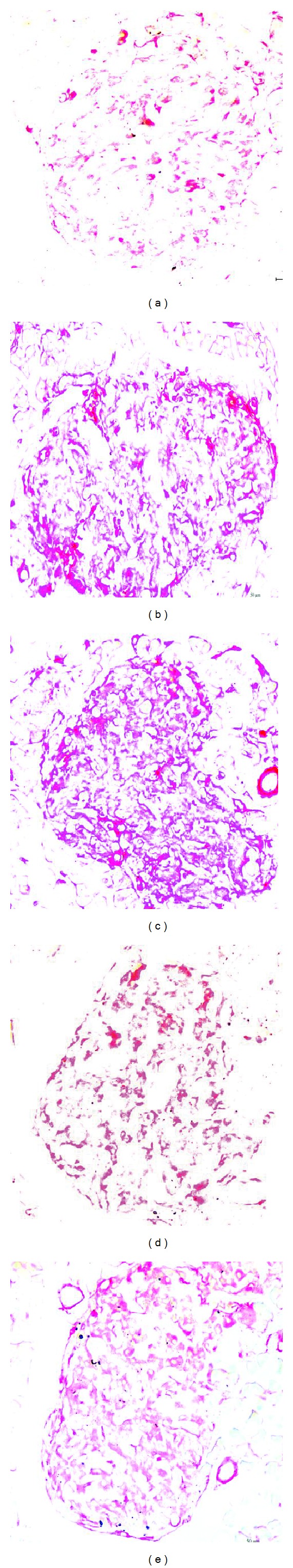
Fibrosis of the pancreatic islets (×400). Fibrosis of the pancreatic islets was significantly increased in MSG rats in the interior area of the islets compared with the Con group. The MSG rats, received HRP treatment had not improve status of islets fibrosis, whereas rats received losartan treatment ameliorate the fibrosis status. (a) Con; (b) MSG; (c) MSG-HRP; (d) MSG-L; (e) MSG-HRP-L.

Fibrosis of the pancreatic islets was evaluated according to picrosirius staining (Figure 6) and the ratio of stained area to the area of whole islets was calculated. The ratio was significantly increased in MSG group (61.5% ± 8.92%) when compared with the Con group (28.36% ± 6.84%). The MSG rats received losartan (34.0 % ± 7.42%) and both losartan and HRP (35.6% ± 6.32%) treatment decreased the ratio obviously when compared with the MSG Group (*P* values were both <0.01), whereas the MSG rats received HRP treatment (53.0% ± 7.56%) had no statistical difference with the MSG Group.

### 3.7. Pancreas Oxidative Stress

**Figure 7:**
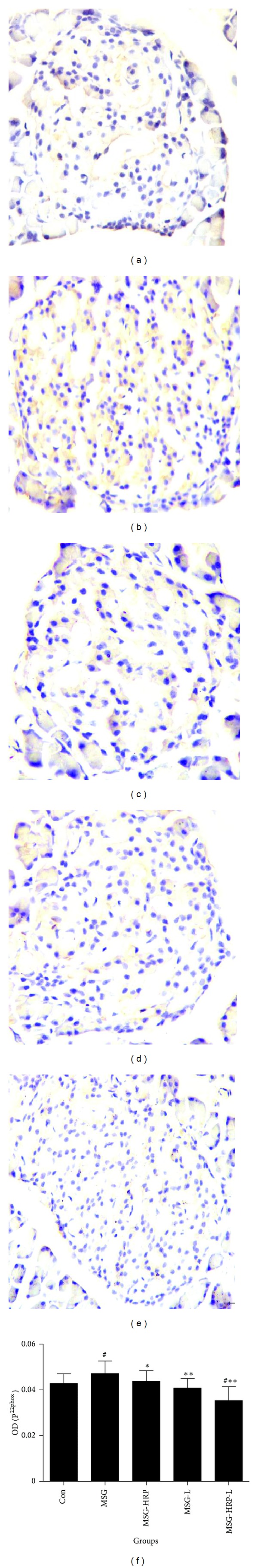
Immunostaining of P^22phox^ in the pancreatic islets ((a)–(e), ×400) and the average gray-scale intensities of staining (f). The immunostaining of P^22phox^ increased in the MSG group when compared with the Con group. Treatment with losartan, HRP, and both decreased the average gray-scale intensities of immunostaining of P^22phox^. (a) Con; (b) MSG; (c) MSG-HRP; (d) MSG-L; (e) MSG-HRP-L. *Compared with MSG group, *P* < 0.05; **compared with MSG group, *P* < 0.01; ^#^compared with Con group, *P* < 0.05.

**Figure 8:**
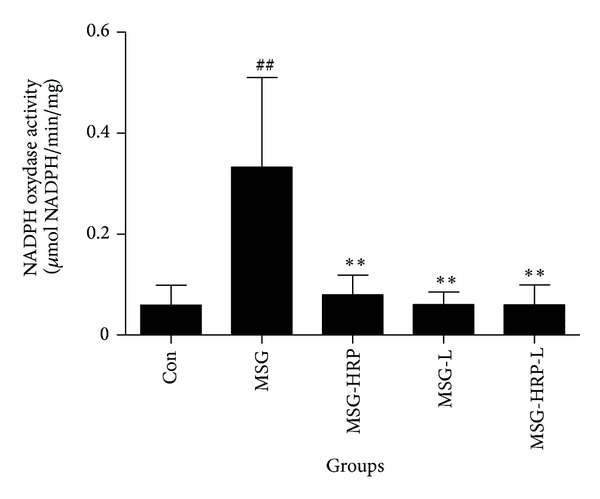
NADPH oxidase activity in pancreatic tissue. **Compared with MSG group, *P* < 0.01; ^##^compared with Con group, *P* < 0.01.

Immunostaining of P^22phox^ in the pancreatic islets in five groups of animals was shown in Figures 7(a)–7(e) and the average gray-scale intensities were shown in Figure 7(f). The immunostaining of P^22phox^ increased in the MSG group when compared with the Con group (*P* < 0.05). Treatment with losartan, HRP, and both decreased the average gray-scale intensities of immunostaining of P^22phox^ (*P* values were <0.05, <0.01, and <0.01, resp.). NADPH oxidase activity in pancreatic tissue was shown in Figure 8. NADPH oxidase activity in pancreatic tissue was increased in MSG rats when compared with the control group. Treatment with losatan, HRP, and both decreased the NADPH oxidase activity in pancreatic tissue (compared with MSG group, *P* values were all <0.01). Increased NADPH oxidase activity was strongly correlated with levels of local Ang-II in pancreatic tissues (*r* = 0.665, *P* < 0.01).

## 4. Discussion

A large injection of sodium L-glutamate into newborn SD rats generated necrosis of neuronal cells of the ventromedial nucleus and arcuate nucleus in the hypothalamus, and as a result, the rats developed polyphagia, obesity, and energy regulation barriers [22–25]. Our results showed that MSG rats given high-energy diet from 3 weeks age to 12 weeks led to obesity, insulin resistance, and elevated blood glucose level after glucose load. However, levels of insulin releasing after glucose load just reached about half of the normal SD rats. The results above demonstrated that MSG rat was a model with insulin resistance and **β**-cell impairment. Nemeroff et al. showed that MSG rats had smaller endocrinic organs such as pituitary, testis, ovarian, adrenal, and lower levels of thyroid hormone [22] and gave us a cue that **β**-cell impairment may be a result of dysplasias of pancreas islets. Although serum insulin concentration was obviously lower than the normal SD rats, the MSG rats had elevated blood glucose after glucose load but kept normal fasting blood glucose at age of 12 weeks and may due to relative low levels of antergic hormone of insulin such as glucocorticoids and thyroid hormone. The characteristic of glucose status of the MSG rats supplied us with an ideal model for the study.

Several key RAS components were detected in the pancreas tissue. Leung et al. showed that the rat pancreas expressed the major RAS component genes, notably angiotensinogen and renin, required for intracellular formation of angiotensin II [26]. Tahmasebi et al. [16] and Lam and Leung [27] testified the presence of the AT1 receptor, AT2 receptor, angiotensinogen and (pro)renin in the human *β* cell of the islets. Our results showed that (pro)renin, angiotensinogen, and (P)RR were detected in pancreatic islets, and the level of local Ang-II was independent of system Ang-II.

The components of local RAS are responsive to various physiological and pathophysiological stimuli such as hyperglycemia and lead to aggravation of islets functions in turn [28–33]. Activation of pancreatic local RAS increased in different type 2 diabetes animal models including db/db mice, ZDF rats, and OLETF rats. Our results showed that level of pancreatic local Ang-II was remarkedly increased in MSG rats. Animal studies indicated that RAS inhibitors improved islets functions. Pretreatment of isolated db/db islets with losartan before the addition of angiotensin II (100 nmoL/L) not only completely rescued glucose-induced insulin secretion but also tended to increase insulin release to an even higher level [31]. Treatment with perindopril or irbesartan treatments significantly improved first-phase insulin secretion in ZDF animals [30]. Ramipril treatment remarkably reduced weight gain and the area under the curve of glucose [28]. Liskiren decreased body weight and plasma glucose level and increased plasma insulin level in a fed condition [34]. Our results showed that losartan increased the levels of insulin releasing after glucose load and decreased AUC of blood glucose. Much to our surprise, HRP improved insulin sensitivity but had not increased insulin releasing and had not improved glucose status.

The maintenance of the specialized architecture of the pancreatic islet and normal *β*-cell mass is important for continuing function. RAS inhibitors increased *β*-cell mass. Candesartan increased *β*-cell mass and increased staining intensity of insulin in pancreas islets of db/db mice [33]. Treatment with aliskiren restored the *β*-cell mass to a similar level to that in nondiabetic normal (C57BL/6J) mice [34]. Our results indicated that losartan increased *β*-cell mass and decreased *α*-cell mass in accordance with the results of insulin releasing test. The regulation of islet cell apoptosis and proliferation is important in maintaining normal ratio of *β*-cell mass and *α*-cell mass. Our results showed PCNA-positive staining cells distributed in the periphery of the islets in accordance with *α* cells in MSG rats. Losartan but not HRP decreased the PCNA-positive staining cell and may be the reason why HRP had not improved glucose status of the MSG rats.

Pancreatic islets are highly susceptible to oxidative injury, owing to low endogenous antioxidant activity. Blockade of the RAS with perindopril or irbesartan significantly reduced staining for nitrotyrosine in ZDF rats [30]. Candesartan decreased staining intensity of components of NAD(P)H oxidase, P^22phox^, gp^91phox^, and those of oxidative stress markers in *β*-cell of db/db mice [33]. Treatment with losartan, HRP, and both decreased the average gray-scale intensities of immunostaining of P^22phox^ and NADPH oxidase activity in pancreatic tissue. The results above supported the notion that RAS inhibitors improve islets functions by decreasing activity of oxidative stress.

Fibrosis is another factor that can change the specialized architecture of the pancreatic islet. Both perindopril and irbesartan reduced expression for collagen I and IV protein in ZDF rats [30]. Islet fibrosis and the expression of TGF-*β* with its downstream signal molecules were significantly reduced in the pancreas of OLETF ramipril-treated group than in control group [28]. Although Ichihara et al. [35, 36] demonstrated that HRP decreased the expression of collagen I and III in the heart and collagen IV in the kidney in spontaneously hypertensive rats, HRP had not improved status of islets fibrosis in MSG rats.

From the above we can conclude that HRP and losartan had some similar effects on islets in MSG rats. Losartan decreased level of local Ang-II, increased *β*-cell mass, and decreased the activity of oxidative stress. Of course, different effects of these two agents were also obvious. HRP had no effect on the *α*-cell mass and proliferation of islet cells and had not improved status of islets fibrosis in MSG rats. These difference may be due to the specially interaction of renin, prorenin, and (P)RR.

Renin is an aspartyl protease that cleaves angiotensinogen into angiotensin I, the rate-limiting reaction in the cascade generating angiotensin. Both renin and its inactive precursor, prorenin, can bind to the (P)RR. The (P)RR is a true receptor that is able to activate intracellular signaling, and (P)RR bound prorenin is enzymatically active as a result of a conformational change without cleavage of the prosegment. As a blocked of (P)RR, HRP decreased local level of Ang-II, and in the end, oxidative stress was decreased as it is Ang-II dependant. However, whether the activation of ERK1/2 MAPK pathway which was related to proliferation and fibrosis was blocked by HRP needs further investigation [37].

## 5. Conclusions

In summary, on the one hand, both losartan and HRP decreased levels of pancreatic local Ang-II and NADPH oxidase activity as well as its subunits P^22phox^. On the other hand, losartan but not HRP decreased *α*-cell mass and number of PCNA-positive cells located periphery of the islets and decreased picrosirius red stained area in islets. HRP ameliorating insulin resistance but not *β*-cell functions leads to hyperglycemia in the end in male MSG rats, and the dual characters of HRP may partly account for the phenomenon.

## Figures and Tables

**Table 1:** Physiological and biochemical parameters of the study groups.

Groups	*n *	Weight (g)	Length (cm)	Lee's index	Celiac adipose adipose tissue mass (mg wet wt./g body wt)	Systolic blood pressure (mmHg)	AT-II (pg/mL)
Con	6	304.60 ± 13.28	23.00 ± 0.71	292.73 ± 10.67	0.019 ± 0.004	137.83 ± 11.62	67.09 ± 10.55
MSG	6	349.60 ± 24.60	21.80 ± 0.45^#^	323.09 ± 9.54^##^	0.073 ± 0.020^##^	151.52 ± 25.62	78.32 ± 12.54
MSG-HRP	6	314.15 ± 47.68	22.00 ± 1.22^#^	308.70 ± 11.59^#^	0.060 ± 0.025^##^	132.29 ± 14.49	60.13 ± 34.28
MSG-L	6	298.8 ± 43.95*	20.9 ± 0.41^##^	319.25 ± 14.15^##^	0.061 ± 0.017^##^	108.34 ± 6.97**	90.03 ± 7.15
MSG-HRP-L	6	297.67 ± 29.79*	21.17 ± 0.52^##^	315.33 ± 13.97^##^	0.057 ± 0.016^##^	108.67 ± 3.84**	119.06 ± 20.47^##∗∗^

*Compared with MSG group, *P* < 0.05; **compared with MSG group, *P* < 0.01.

^#^Compared with Con group, *P* < 0.05; ^##^compared with Con group, *P* < 0.01.

Lee's index = $\sqrt[{3}]{\text{Weight }\left(\text{g}\right)}\times 1000/\text{Length }\left(\text{cm}\right)$.
